# Prevalence and clinical associations of anti-rods and rings antibodies in ANA-tested patients

**DOI:** 10.1007/s12026-026-09754-6

**Published:** 2026-02-19

**Authors:** Baris Can, Arzu Aksit Ilki

**Affiliations:** Department of Medical Microbiology, Marmara University School of Medicine, Basıbuyuk Street No: 9 D:2, Istanbul, Maltepe 34854 Turkey

**Keywords:** ANA, Anti-rods and rings, Indirect immunofluorescence, Autoantibodies, Autoimmune diseases

## Abstract

Anti-rods and rings (anti-RR) antibodies represent a rare cytoplasmic antinuclear antibody (ANA) pattern typically associated with hepatitis C virus (HCV) infection and interferon-based therapy. However, emerging evidence suggests a broader clinical relevance beyond HCV. This study aimed to investigate the prevalence, immunofluorescence characteristics, autoantibody associations, and clinical contexts of anti-RR antibodies in a large Turkish patient cohort undergoing routine ANA screening. Serum samples were retrospectively analysed for ANA using indirect immunofluorescence (IIF) between January 2022 and October 2024. Samples displaying anti-RR patterns were further evaluated for specific ANA profiles using line immunoassay. Clinical and demographic data were collected from medical records. Among 57,644 patients tested for ANA, 11,752 (20.39%) were ANA-positive, with only 91 cases (0.16%) exhibiting an anti-RR pattern. These cases were predominantly female (60.44%) with a median age of 52 years. Isolated anti-RR patterns were observed in 70.33% of patients, while the remainder showed mixed patterns, most commonly with AC-4,5,31 and AC-1 ANA patterns. Anti-RR positivity showed no significant association with classical ANA-specific autoantibodies. Clinically, anti-RR-positive patients displayed diverse diagnoses, including autoimmune diseases (21.98%), nephropathic conditions (9.89%), hepatic (7.69%) and pulmonary (6.59%) disorders, with the majority (53.85%) categorized under other conditions. Anti-RR antibodies, while rare, occur across a spectrum of diseases and may be associated with heterogeneous clinical conditions beyond hepatitis C infection. Their detection warrants careful interpretation within the appropriate clinical and laboratory context. Prospective studies are needed to explore their pathophysiological roles and diagnostic utility in diverse patient populations.

**Supplementary Information:**

The online version contains supplementary material available at 10.1007/s12026-026-09754-6.

## Introduction

Antinuclear antibodies (ANAs) are a hallmark in the diagnosis of systemic autoimmune diseases such as systemic lupus erythematosus (SLE), systemic sclerosis, and Sjögren’s syndrome. ANA testing via indirect immunofluorescence (IIF) on HEp-2 cells reveals a spectrum of nuclear, cytoplasmic, and mitotic patterns that provide diagnostic clues. Among these, the ‘rods and rings’ (RR) pattern is a rare cytoplasmic phenomenon. According to the International Consensus on ANA Patterns (ICAP), anti-rods and rings antibodies correspond to the AC-23 cytoplasmic pattern, which can only be reliably detected on HEp-2 cell substrates provided by selected manufacturers.

First reported in patients undergoing pegylated-interferon and ribavirin treatment for chronic HCV infection, anti-RR antibodies produce distinctive rod-like (3–10 μm) or ring-like (2–5 μm) structures under fluorescence microscopy. The primary antigenic components of these structures have been identified as inosine monophosphate dehydrogenase 2 (IMPDH2) and cytidine triphosphate synthase 1 (CTPS1) enzymes essential for guanine and cytidine nucleotide biosynthesis [1].

Recent studies suggest a broader context for anti-RR antibodies, implicating them in conditions beyond HCV, potentially reflecting cellular metabolic disturbances or drug-induced stress [2–4]. Given these emerging insights, a comprehensive evaluation of anti-RR prevalence and associated clinical scenarios is warranted.

## Materials and methods

This retrospective cohort study analysed 57,644 consecutive ANA test results performed between January 2022 and October 2024 in a tertiary care training and research hospital in Turkey. ANA screening was performed via IIF using HEp-2 cell and primate liver substrates (EUROIMMUN, Germany), starting with a 1:100 serum dilution and continuing with serial dilutions. Fluorescent patterns were examined under the fluorescence microscope. Anti-RR patterns were identified and validated by experienced microbiologists in accordance with the International Consensus on ANA Patterns (ICAP; www.anapatterns.org) guidelines.

ANA-positive samples underwent additional evaluation using line immunoassay (EUROLINE, Germany) for the detection of specific autoantibodies (anti-nRNP/Sm, anti-Sm, anti-SS-A, anti-Ro-52, anti-SS-B, anti-Scl-70, anti-PM-Scl-100, anti-Jo-1, anti-CENP-B, anti-PCNA, anti-dsDNA, anti-nucleosome, anti-histone, anti-ribosomal P-protein, anti-AMA-M2 and anti-DFS70). Clinical data were obtained from the hospital information management system, and diagnoses were grouped into predefined categories (autoimmune, nephropathic, hepatic, pulmonary, etc.).

## Results

**Fig. 1:**
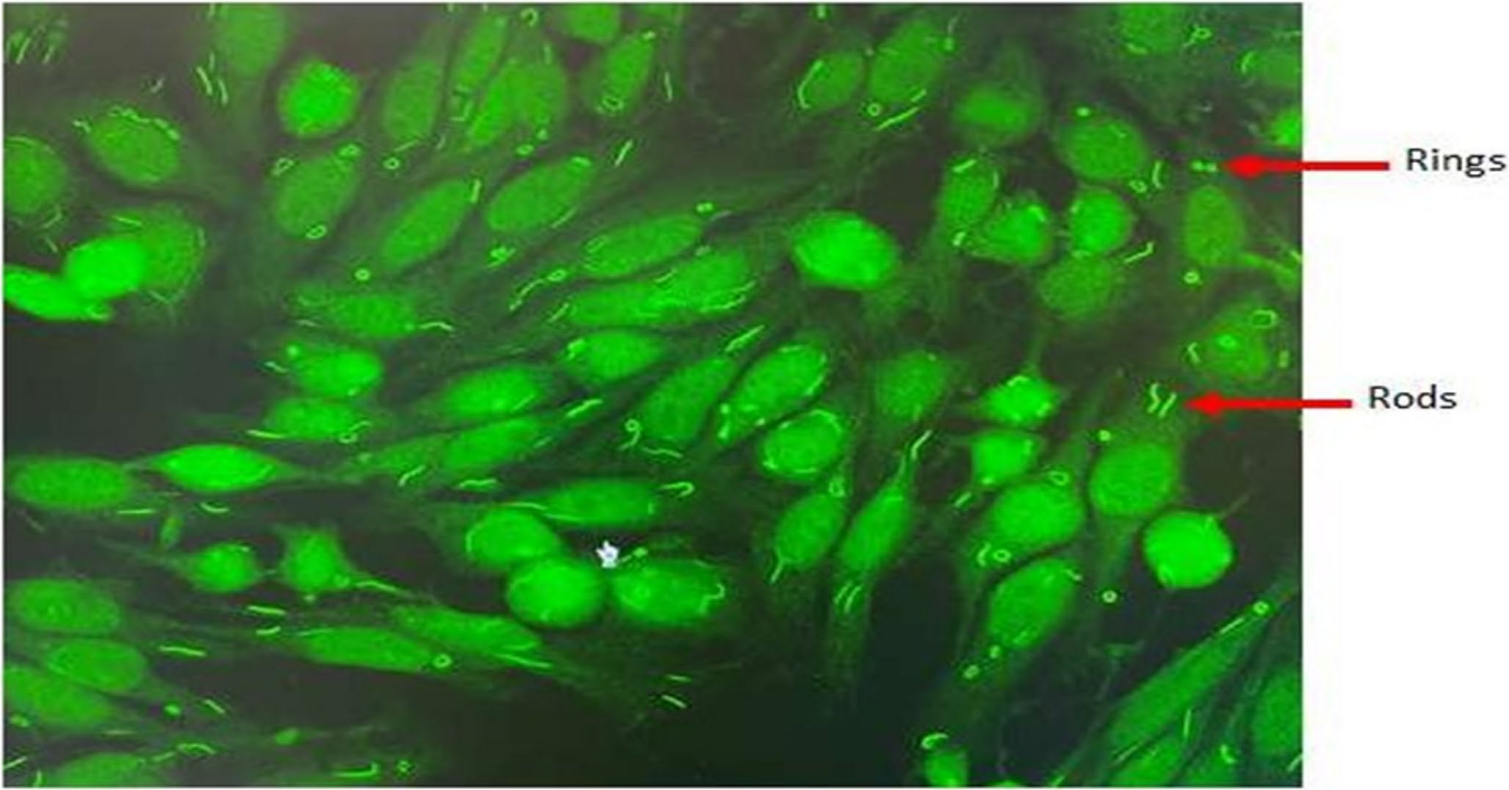
Representative anti-RR immunofluorescence in HEp-2 cells showing cytoplasmic rod-like (3–10 μm) and ring-like (2–5 μm) structures at 1:100 dilution (IIF). Image generated by the authors using representative patient samples

Out of 57,644 serum samples tested, 11,752 (20.39%) were positive for ANA, and only 91 of these (0.16%) showed anti-RR patterns. Most of the patients with anti-RR positivity were females (60.44%), and the median age was 52 years. Clinical department distribution revealed that patients most frequently presented to rheumatology (34.06%), followed by neurology (15.39%) and internal medicine (12.09%). Other departments included physical medicine and rehabilitation (9.89%) and nephrology (7.69%), with the remaining 20.88% categorized as others. Notably, 70.33% of patients exhibited isolated anti-RR patterns without concurrent ANA patterns, whereas 29.67% demonstrated mixed ANA patterns, most frequently AC-4,5,31 (62.97%), followed by AC-1 (25.93%) (Table 1). A representative immunofluorescence image of the anti-RR pattern observed in our study is shown in Fig. 1. This distribution highlights the potential for anti-RR antibodies to occur both independently and in combination with other ANA patterns.

In the subgroup of patients with anti-RR positivity, specific ANA-associated autoantibodies were infrequently detected. The most commonly identified autoantibody was anti-SS-A, present in 5 patients (5.49%), followed by anti-Jo-1 and anti-Ro-52, each observed in 3 patients (3.30%). Other autoantibodies, including anti-Scl-70, anti-dsDNA, anti-DFS70, anti-CENP-B, anti-RNP/Sm, and anti-SS-B, were each detected in only one patient (1.10%). This low rate of concurrent autoantibody positivity supports the observation that anti-RR patterns frequently occur in isolation and are not strongly associated with classical systemic autoimmune profiles (Table 1).

**Table 1:** Demographic and clinical data in patients with anti-RR positivity (*n* = 91)

		Number (n)	Percent (%)
Gender	Female	55	60.44%
	Male	36	39.56%
Clinics Applied	Rheumatology	31	34.06%
	Neurology	14	15.39%
	Internal Medicine	11	12.09%
	Physical Medicine and Rehabilitation	9	9.89%
	Nephrology	7	7.69%
	Others	19	20.88%
Concomitant ANA Positivity	Negative	64	70.33%
	Positive	27	29.67%
ANA-associated autoantibodies (n:91)	Anti-SS-A	5	5.49%
	Anti-Jo-1	3	3.30%
	Anti-Ro-52	3	3.30%
	Anti-Scl-70	2	2.20%
	Anti-dsDNA	1	1.10%
	Anti-DFS70	1	1.10%
	Anti-CENP-B	1	1.10%
	Anti-RNP/Sm	1	1.10%
	Anti-SS-B	1	1.10%
Concomitant ANA pattern positivity (n:27)	AC-1	7	25.93%
	AC-2	1	3.70%
	AC-4,5,31	17	62.97%
	AC-6	1	3.70%
	AC-8	1	3.70%

The clinical spectrum of anti-RR-positive individuals encompassed a broad range of systemic diseases. Autoimmune conditions were present in 21.98% of cases, with rheumatoid arthritis (35.00%), Sjögren’s syndrome (20.00%), and ankylosing spondylitis (15.00%) being the most prevalent. Renal involvement was noted in 9.89% of patients, primarily as renal insufficiency or failure. Hepatic conditions accounted for 7.69% of cases, including hepatitis C (42.85%) and hepatitis B (28.57%). Pulmonary diseases, such as pulmonary embolism and interstitial lung disease, were observed in 6.59% of patients. Nearly half of the patients (53.85%) were categorized under other conditions, of whom arthropathies were the most frequent diagnosis (44.90%). A majority (79.12%) had a history of drug use, most commonly immunosuppressants (13.89%), hydroxychloroquine (12.50%), corticosteroids (11.11%), and colchicine (6.94%). Only a small proportion (4.17%) had received pegylated interferon and ribavirin, suggesting that anti-RR antibodies may be associated with broader clinical contexts beyond classical HCV therapy (Table 2).

**Table 2:** Disease and drug history of anti-RR-positive patients (*n* = 91)

		*Number (n)*	*Percent (%)*
Autoimmune Diseases (n:20)	Rheumatoid arthritis	7	35.00%
	Sjögren’s syndrome	4	20.00%
	Ankylosing Spondylitis	3	15.00%
	Systemic scleroderma	2	10.00%
	Other Autoimmune Diseases^a^	4	20.00%
Nephropathy (n:9)	Renal insufficiency	4	44.44%
	Renal failure	3	33.33%
	Proteinuria	1	11.11%
	Nephrotic Syndrome	1	11.11%
Hepatic diseases (n:7)	Hepatitis C	3	42.85%
	Hepatitis B	2	28.57%
	Toxic Hepatitis	1	14.29%
	Hepatic cirrhosis (Cryptogenic)	1	14.29%
Pulmonary diseases (n:6)	Pulmonary Embolism	3	50.00%
	Pulmonary interstitial diseases	2	33.33%
	Pulmonary infection	1	16.67%
Other diseases (n:49)	Arthropathy^d^	22	44.90%
	Dermatosis^e^	7	14.29%
	Endocrine diseases^f^	7	14.29%
	Familial Mediterranean Fever	3	6.12%
	Undefined diseases^g^	10	20.41%
Use of Drugs (n:72)	Immunosuppressive drugs^b^	10	13.89%
	Hydroxychloroquine	9	12.50%
	Corticosteroid	8	11.11%
	Colchicine	5	6.94%
	PEG-IFN/RBV^c^	3	4.17%
	Other drugs	37	51.39%

Footnotes:

^a^ Other autoimmune diseases: autoimmune hepatitis, undifferentiated connective tissue disease, Crohn’s disease, psoriatic arthritis

^b^ Immunosuppressive drugs: methotrexate, cyclophosphamide, azathioprine

^c^ PEG-IFN/RBV: pegylated interferon plus ribavirin combination therapy

^d^ Arthropathy: joint pain and arthritis

^e^ Dermatosis: urticaria, vitiligo, dermatitis, rash, psoriasis, alopecia

^f^ Endocrine diseases: hypertension, diabetes mellitus, thyroid disease

^g^ Undefined diseases: ascites, abdominal infection, gastroenteritis, coronary heart disease, cardiopathy, ventricular failure, arrhythmia, cerebrovascular disease

## Discussion

The prevalence of anti-rods and rings (anti-RR) antibodies observed in this study (0.16% of ANA-positive samples) supports the well-established notion that these antibodies are rare among patients undergoing ANA testing. Comparable rates have been reported globally, including in Dalian, China (0.20%) [5] and in other large retrospective cohorts [6]. These findings confirm that anti-RR, while uncommon, represents a distinct cytoplasmic ANA pattern of potential diagnostic interest. Historically, anti-RR antibodies were closely linked to hepatitis C virus (HCV) infection, particularly among patients undergoing pegylated interferon (PEG-IFN) and ribavirin therapy [7–9]. However, recent evidence suggests that anti-RR positivity extends beyond this narrow association [10, 11]. Our study detected anti-RR antibodies in patients with a broad spectrum of autoimmune, renal, hepatic, and pulmonary diseases. This broader clinical spectrum is consistent with findings from Turkey [12], where similar variability was observed. In terms of underlying mechanisms, anti-RR antibodies target cytoplasmic filamentous structures composed of inosine monophosphate dehydrogenase type 2 (IMPDH2) and cytidine triphosphate synthase type 1 (CTPS1), two key enzymes in nucleotide biosynthesis [1]. Pharmacological agents such as ribavirin, azathioprine, and mycophenolic acid are known to induce structural modifications in these enzymes, potentially leading to the formation of immunogenic rods and rings [13, 14].

In our cohort, a substantial majority of anti-RR-positive patients (79.12%) had a history of polypharmacy, including notable frequencies of immunosuppressants (13.89%), hydroxychloroquine (12.50%), corticosteroids (11.11%) and colchicine (6.94%) (Table 2). Only a small subset (4.17%) received pegylated interferon and ribavirin, the classical anti-RR inducing regimen historically associated with HCV therapy [8, 15, 16]. This distribution supports the hypothesis that anti-RR antibody formation is not limited to HCV or IFN/RBV exposure.

In some studies, anti-RR antibodies have been proposed as a human model of drug-induced autoantibody generation, showing that ribavirin, and other inhibitors of nucleotide biosynthesis such as mycophenolic acid, can induce rods and rings structures and promote subsequent autoantibody production in vitro and in vivo [17]. Further studies demonstrated the differential capacity of such pharmacological agents to induce RR structures, highlighting the possibility that diverse drug classes may contribute to this phenomenon [18]. Moreover, large-scale population data from NHANES indicated that anti-RR antibodies can occur in apparently healthy individuals with no history of HCV infection or IFN/RBV use; many of these individuals exhibited polypharmacy, suggesting broader pharmacological triggers beyond viral therapy [11].

Overall, these findings support a model wherein several immunomodulatory or cytotoxic medications, including but not limited to ribavirin, azathioprine, mycophenolic acid, or corticosteroids may initiate structural perturbations in enzymes such as IMPDH2 and CTPS1, thereby facilitating anti-RR generation. In our cohort, the predominance of non-IFN/RBV medication exposure aligns with this broader conceptual framework, suggesting that anti-RR may be a serological marker of pharmacologically induced metabolic stress rather than a niche byproduct of hepatitis C treatment alone.

Importantly, in our cohort, anti-RR antibodies showed no correlation with classical ANA-specific autoantibodies (e.g., anti-dsDNA, anti-SSA/anti-Ro, anti-Scl-70). These findings align with international standardization efforts by the International Consensus on ANA Patterns (ICAP), which recognizes anti-RR as a distinct cytoplasmic pattern (AC-23) in ANA testing [19]. In addition, recent studies suggest that anti-RR antibodies may have a potential role in metabolism. For instance, associations with altered triglyceride levels and renal function have been described, implying broader systemic implications [10, 20]. Overall, anti-RR antibodies appear to represent not only a serological marker of certain treatment exposures (e.g., ribavirin) but also a broader indicator of cellular stress, altered nucleotide metabolism, or immune dysregulation. Their detection, though rare, should prompt clinicians to consider both hepatic and non-hepatic disease contexts. Prospective studies integrating immunological, metabolic, and pharmacogenomic approaches will be critical in fully elucidating their pathophysiological significance.

Although anti-RR antibodies were frequently detected as isolated ANA patterns, the current evidence does not support their use as an entry criterion for the classification of systemic lupus erythematosus, given their low prevalence and lack of disease specificity. Given their very low prevalence, substrate dependency, and lack of consistent association with defined disease entities, anti-RR antibodies should be reported as a rare cytoplasmic ANA pattern with limited standalone diagnostic value; laboratories should clearly state the substrate used, and clinicians should interpret this finding only in conjunction with the full clinical and laboratory context.

This study has several important limitations. First, its retrospective design prevented re-analysis of archived sera on HEp-2 substrates from different manufacturers, despite the known substrate dependency of the AC-23 pattern. Second, the absence of a healthy control group precludes estimation of the background prevalence of anti-RR antibodies in the general population. Third, all ANA testing was performed using a single commercial HEp-2 substrate, which limits generalizability. Finally, longitudinal clinical follow-up data were not available, restricting interpretation of the temporal relationship between anti-RR positivity, drug exposure and clinical outcomes.

## Conclusion

Anti-RR antibodies are rare cytoplasmic ANA patterns that may occur in a wide variety of clinical conditions beyond hepatitis C infection and its treatment. However, due to their very low prevalence, substrate dependency and lack of consistent association with specific diseases, their standalone diagnostic value appears limited. Anti-RR positivity should therefore be interpreted cautiously and in conjunction with the full clinical and laboratory context rather than as a disease-specific marker. Further prospective studies including healthy control populations and multi-manufacturer HEp-2 substrates are required to clarify their clinical significance.

## Supplementary Information

Below is the link to the electronic supplementary material.

Supplementary Material 1 (PDF 867 KB)

## Data Availability

The data supporting the findings of this study are derived from patient records and are not publicly available due to ethical and privacy restrictions. Data may be available from the corresponding author upon reasonable request, subject to institutional approvals.
