# Supplementary material for: Prevalence and clinical associations of anti-rods and rings antibodies in ANA-tested patients
Source: Immunol Res. 2026 Feb 19;74(1):16. doi: 10.1007/s12026-026-09754-6 (PMC12920392; doi:10.1007/s12026-026-09754-6)
Supplement: Supplementary file 1 — Supplementary Material 1 (PDF 867 KB) [file 12026_2026_9754_MOESM1_ESM.pdf]

Marmara Üniversitesi Tıp Fakültesi
İlaç ve Tıbbi Cihaz Dışı Araştırmalar Etik Kurulu

	**PROTOKOL KODU**	**09.2024. 561**
	**PROJE ADI**	**Halkalar ve Çubuklar (Rings and Rods) Paterninin Hepatiti B, Hepatit C ve Çeşitli Hastalıkların Tanısındaki Yerinin Değerlendirilmesi**
	SORUMLU ARAŞTIRICI ÜNVANI/ADI	**Uzm. Dr. Barış CAN**

KARAR BİLGİLERİ	**Tarih. 22.04.2024**<br>Yukarıda başvuru bilgileri verilen araştırma başvuru dosyası ve ilgili belgeler araştırmanın gerekçe, amaç, yaklaşım ve yöntemleri dikkate alınarak incelenmiş ve gerçekleştirilmesinde sakınca bulunmadığı için Kurulumuzca onaylanmasına oy birliği ile karar verilmiştir. Onay sonrasında yapılacak her türlü proje değişiklikleri (katılımcılar, başlık vb.) veya protokol değişikliklerinin Etik Kurula bildirilerek proje onayının yenilenmesi gerekmektedir.

ASİL ÜYELER

Unvanı / Adı / Soyadı	Uzmanlık Dalı	Kurumu / EK Üyeliği	Onaylanan Proje ile İlişkisi	Toplantıya katılım	İmza
Prof. Dr. Medine GÜLÇEBİ İDRİZ [illegible]LU	Tıbbi Farmakoloji	M. Ü. Tıp Fakültesi/ Başkan	☐ Var ☐ Yok	☐ Evet ☐ Hayır	
Doç. Dr. Fethi GÜL	Anesteziyoloji ve Reanimasyon	M. Ü. Tıp Fakültesi/Başkan Yrd.	☐ Var ☐ Yok	☐ Evet ☐ Hayır	
Prof. Dr. Dilşad SAVE	Halk Sağlığı	M. Ü. Tıp Fakültesi/Üye	☐ Var ☐ Yok	☐ EVET ☐ HAYIR	
Prof. Dr. Gürkan SERT	Tıp Tarihi ve Etik	M. Ü. Tıp Fakültesi Üye	☐ Var ☐ Yok	☐ Evet ☐ Hayır	
Prof. Dr. Neşe PERDAHLI FİŞ	Çocuk ve Ergen Ruh Sağlığı ve Hast.	M. Ü. Tıp Fakültesi Üye	☐ Var ☐ Yok	☐ Evet ☐ Hayır	
Doç. Dr. Osman KÖSTEK	Tıbbi Onkoloji	M. Ü. Tıp Fakültesi/Üye	☐ Var ☐ Yok	☐ Evet ☐ Hayır	
Dr. Öğr. Üyesi Muhammed Hasan TOPER	Tıbbi Patoloji	M. Ü. Tıp Fakültesi/Üye	☐ Var ☐ Yok	☐ Evet ☐ Hayır	
Dr. Öğr. Üyesi Can ERZİK	Tıbbi Biyoloji	M. Ü. Tıp Fakültesi/Üye/Raportör	☐ Var ☐ Yok	☐ Evet ☐ Hayır	
Sevinç YAŞAR	Sağlık Mensubu olmayan kişi	Dış Üye	☐ Var ☐ Yok	☐ Evet ☐ Hayır	
Prof. Dr. Berna TERZİOĞLU [illegible]EBİTOĞLU	Tıbbi Farmakolji	M. Ü. Tıp Fakültesi/Yedek Üye	☐ Var ☐ Yok	☐ Evet ☐ Hayır	
Prof. Dr. Mustafa Ümit UĞURLU	Genel Cerrahi	M. Ü. Tıp Fakültesi/Yedek Üye	☐ Var ☐ Yok	☐ Evet ☐ Hayır	
Prof. Dr. Seyhan HIDIROĞLU	Halk Sağlığı	M. Ü. Tıp Fakültesi/Yedek Üye	☐ Var ☐ Yok	☐ Evet ☐ Hayır	
Av. Seda TAN	Hukukçu	Dış Üye	☐ Var ☐ Yok	☐ Evet ☐ Hayır	
Doç. Dr. Burcu HİŞMİ ÖZTÜRK	Çocuk Sağlığı ve Hast.	M. Ü. Tıp Fakültesi/Yedek Üye	☐ Var ☐ Yok	☐ Evet ☐ Hayır	
Doç. Dr. İbrahim Vedat BAYOĞLU	Tıbbi Onkoloji	M. Ü. Tıp Fakültesi/Yedek Üye	☐ Var ☐ Yok	☐ Evet ☐ Hayır	
Doç. Dr. Necati ENVER	Kulak, Burun ve Boğaz Hast.	M. Ü. Tıp Fakültesi/Yedek Üye	☐ Var ☐ Yok	☐ Evet ☐ Hayır	
Doç. Dr. Banu AYDIN OMAY	Biyofizik	M. Ü. Tıp Fakültesi/Yedek Üye	☐ Var ☐ Yok	☐ Evet ☐ Hayır	
Özlem KÜSKÜ	Sağlık Mensubu olmayan kişi	Dış Üye	☐ Var ☐ Yok	☐ Evet ☐ Hayır	
